# Editorial: Public health promotion and medical education reform, volume II

**DOI:** 10.3389/fpubh.2023.1267528

**Published:** 2023-08-15

**Authors:** Jing Tian, Jian Chen

**Affiliations:** School of Basic Medical Sciences, Guilin Medical University, Guilin, China

**Keywords:** public health, medical education reform, public health education, trends and challenges, local/regional medical schools

During the COVID-19 pandemic, all the involved countries have to face the tough challenges from overloaded public health system, and thus have been taking various measures to strengthen the system (1, 2). Indeed, since the onset of COVID-19, the public healthcare system has underwent profound changes in so many ways such as: education, medical research, public health infrastructure, health policy and management (3, 4).

One of the thorniest issues that all countries have to solve is the shortage of frontline healthcare workers, which is even more worsen in local and rural places where the density of healthcare workers always decreases with deprivation (5). Consequently, the frontline medical workers endured extra duty hours, workload burden and health risks during the novel coronavirus crisis (6). This, coupled with the direct engagement with coronavirus infected patients, led the physicians to experience significant stress, producing noticeable psychological alterations (7). Guraya et al. evaluated the impact of COVID-19 outbreak on the frontline physician's psychological health, which was represented in five themes: mental health, individual challenges, decision-making, patient care and support services. Among them, the mental health ranked the foremost challenge to physicians. When the physician's personal and professional lives were highly affected during the COVID-19 pandemic, emotional distress has been witnessed in most of clinical physicians, thereby worsening their psychological condition and preventing them from serving in the patients' best interest (8). Meanwhile, there is plenty of evidence that the priorities for medical care have changed dramatically. Physicians were more likely to consider age, cognitive status, and the prognosis for survival in making decision (9). In addition, there are indications of a paradigm change in the pattern of patient care during the COVID-19 pandemic. That is, accompanied with focus on minimizing infection spread, a patient's care was often delayed and compromised, along with poor patient-doctor communication. On the other hand, the institutional interventions were not adequate to support doctors' crumbling wellbeing. Together, during the pandemic and even in the post-pandemic of COVID-19, the deteriorating mental health of frontline doctors should be paid adequate attention and supported effectively.

In spite of the corresponding increase in the overall density of doctors, the insufficient quantity and quality still failed to support health care demand, particularly in local and rural places, leading to relatively lagged construction of healthcare system (10). The National Development and Reform Commission of China launched the rural-oriented tuition-waived medical education (RTME) program in March 2010 (11). In the recent decade, over 57,000 rural-oriented tuition-waived medical students (RTMSs) were enrolled and received financial aid from government (12). In turn, these students were committed to work in rural medical institutions for at least 3 years after graduation. However, the policy implementation did not achieve expected goals. Zhang et al. reported that less than half of students had strong willing to fulfill the commitments, while the rest may not continue working in rural medical institutions after 3 year-rural service. This would greatly impede the development of China's rural medical system, which makes it unable to mount a quick and effective response to the threat of a pandemic such as COVID-19. Further analysis indicated that the honesty-credit, specialty identity and career identity could significantly predict RTMSs' willingness to fulfill the contract early. Therefore, in order to ensure the sustainability and success of RTME program, students should be encouraged to choose their majors voluntarily, and career identity education should be introduced in the curriculum. Last but not least, with full support from the state and the government, the improved working conditions of rural health workers may encourage RTMSs to stay in rural communities.

As we know, the COVID-19 lockdown pushed the switch from offline to online teaching (13, 14). Then, an important question that remains to be approached is whether this switch would influence education outcomes. Ettl et al. evaluated the feasibility and effectiveness of an online Moodle course in teaching cardiopulmonary resuscitation. It was demonstrated that e-learning method enabled students to gain objective and subjective knowledge, and the interactive learning videos were well-received by students as helpful to their learning. Actually, there is increasing evidence that e-learning may be a reliable alternative, and the online learning has been growing to play an increasingly important role in higher education (15, 16).

Noteworthy, the outbreak of global acute infectious disease such as SARS, H1N1 and COVID-19 has raised high concerns in emergency management and emergency response (17). However, the lack of public health knowledge could be observed in most clinicians (18). Yu et al. reported that, due to the separation of clinical and preventive education systems in current undergraduate medical education, the clinical medicine graduates were not well versed in public health emergency (PHE) practices, while the preventive graduates were lack of medical rescue knowledge and practical capabilities. It was suggested that a complete integrated PHE curriculum should be added to the school's teaching plans regardless of majors.

At the same time, COVID-19 revealed the urgent need to bridge the prominent gap between new knowledge and evidence-based practices. Today's reality is that healthcare professionals are required to possess competency in designing, implementing, and diffusing evidence-based healthcare solutions (19). Since 2015, the Indiana University has begun to offer a Professional Certificate Program as a graduate course, and succeeded in training well-rounded members in innovation and implementation science (20). Mehta et al. stated that the program has built an integrated network of Agile Change Conductors who are competent in developing effective teams, implementing and diffusing evidence-based care within the healthcare delivery system.

In summary, the COVID-19 pandemic has widely impacted public health, specifically focusing on education and training of the healthcare workforce. Learning from the experience of the COVID-19 pandemic will be an effective strategy to contain the spread of future global crises. Undoubtedly, the medical and public health systems would be developed best after combating COVID-19.

## Author contributions

JC: Writing—review and editing. JT: Writing—original draft.
